# Masked Priming of Conceptual Features Reveals Differential Brain Activation during Unconscious Access to Conceptual Action and Sound Information

**DOI:** 10.1371/journal.pone.0065910

**Published:** 2013-05-31

**Authors:** Natalie M. Trumpp, Felix Traub, Markus Kiefer

**Affiliations:** Department of Psychiatry, University of Ulm, Ulm, Germany; University of California, San Francisco, United States of America

## Abstract

Previous neuroimaging studies suggested an involvement of sensory-motor brain systems during conceptual processing in support of grounded cognition theories of conceptual memory. However, in these studies with visible stimuli, contributions of strategic imagery or semantic elaboration processes to observed sensory-motor activity cannot be entirely excluded. In the present study, we therefore investigated the electrophysiological correlates of unconscious feature-specific priming of action- and sound-related concepts within a novel feature-priming paradigm to specifically probe automatic processing of conceptual features without the contribution of possibly confounding factors such as orthographic similarity or response congruency. Participants were presented with a masked subliminal prime word and a subsequent visible target word. In the feature-priming conditions primes as well as targets belonged to the same conceptual feature dimension (action or sound, e.g., typewriter or radio) whereas in the two non-priming conditions, either the primes or the targets consisted of matched control words with low feature relevance (e.g., butterfly or candle). Event-related potential analyses revealed unconscious feature-specific priming effects at fronto-central electrodes within 100 to 180 ms after target stimulus onset that differed with regard to topography and underlying neural generators. In congruency with previous findings under visible stimulation conditions, these differential subliminal ERP feature-priming effects demonstrate an unconscious automatic access to action versus sound features of concepts. The present results therefore support grounded cognition theory suggesting that activity in sensory and motor areas during conceptual processing can also occur unconsciously and is not mandatorily accompanied by a vivid conscious experience of the conceptual content such as in imagery.

## Introduction

Concepts stored in human semantic long-term memory [1] provide the cognitive basis for language, action planning and thought [2], [3] because they code the meaning of objects, events and abstract ideas. It is well accepted that concepts represent information about their referents derived from various sensory modalities (e.g., visual, acoustic) as well as from motor actions [4]. The precise nature of conceptual representations in semantic memory, however, is controversially discussed. More recent modality-specific approaches postulate an essential grounding of concepts in perception and action. These grounded or embodied cognition theories [4]–[9] suggest that access to concepts is based on a partial reinstatement of brain activity in modality-specific brain areas, which typically process sensory- and action-related information [10], [11]. Modality-specific theories challenge classical views of conceptual memory that assume an amodal coding of conceptual knowledge distinct from the sensory and motor brain systems [12]–[15]. According to these amodal theories, original modality-specific sensory-motor information is transformed into a common abstract representation format.

Various behavioural [16], [17], neuropsychological [18], [19], electrophysiological [20], [21] and neuroimaging studies [10], [22] support grounded cognition theories by providing evidence for the involvement of modality-specific cortex in conceptual processing. For example, neuropsychological studies showed that patients with lesions affecting the motor system have specific impairments in processing action-related verbs [23], [24]. Furthermore, functional magnetic resonance imaging (fMRI) studies in healthy volunteers revealed that listening to words or sentences referring to actions executed with the mouth, hand or leg activated left-lateralized fronto-parieto-temporal brain systems, which were also involved in action execution [10], [25]–[27]. In event related potential (ERP) recordings, action-related words elicited relatively more positive ERPs at fronto-central electrodes compared with control words [20], [21], [28].

Similar to the representation of action-related conceptual features in the motor cortex, sensory conceptual features are assumed to be represented in the corresponding sensory brain areas (see, for instance, [29], [30]). A functional link between the auditory and conceptual brain systems has been shown in a combined fMRI/ERP study by Kiefer and colleagues [11]: Partially overlapping activation of sound perception and conceptual processing of sound-related concepts was found in left posterior superior and middle temporal gyri (pSTG/MTG), which form a part of auditory association cortex. ERP recordings in this study revealed that words with high versus low relevance of acoustic conceptual features elicited a more negative scalp potential at fronto-central electrodes. ERP effects related to acoustic and action features thus exhibit opposite polarities. Investigating a patient with a focal lesion in left pSTG/MTG, Trumpp and colleagues [18] additionally provided strong evidence for the necessity of this part of auditory association cortex in perceptual as well as in conceptual sound processing: Patient JR was consistently impaired in conceptual processing of sound-related objects as well as in recognizing corresponding sounds across four different experimental tasks. The conclusion of a feature-specific representation of action- and sound-related conceptual information in or close to modality-specific cortex has also been confirmed by a recent fMRI study [31], in which action- and sound-related concepts activated different portions of pSTG/MTG close to motion-sensitive and auditory brain areas, respectively.

The specificity of sensory-motor brain activity for conceptual processing of different conceptual categories has been substantiated in feature-specific repetition priming experiments. Repetition priming, in which the same stimulus (e.g., word) is presented twice, generally results in a reduction of reaction time (behavioral facilitation). Brain activity sometimes increases (repetition enhancement), particularly in response to unfamiliar visible stimuli [32], [33], but most commonly reduces (repetition suppression) along the entire processing chain in perceptual, semantic and response-related areas compared to a control condition without repetition [34]. Thus, if neurophysiological repetition priming effects vary as a function of conceptual feature type, these feature-related differences must arise from differentially pre-activated semantic representations because visual input (visual letter string) and the required response (e.g., reading, lexical decision) are comparable for both word categories. For instance, Kiefer [20] investigated the impact of stimulus repetition on ERPs to words denoting action-related (artifacts) or vision-related concepts (natural kinds). Results revealed feature-specific ERP effects at fronto-central (action-related) and occipito-parietal (vision-related) electrode sites, which were specifically reduced by stimulus repetition. These findings validate feature-related brain activations as neurophysiological reflections of semantic memory organization and strengthen grounded cognition theories of conceptual representations.

Although previous studies indicated that sensory-motor activity reflects access to conceptual features, contribution of imagery [35] or semantic elaboration processes [36] to sensory-motor activity cannot be entirely excluded because stimuli used in these previous studies have been presented consciously perceivable. Please note that according to grounded cognition theory, activity in sensory and motor areas during conceptual processing can also occur unconsciously and is not mandatorily accompanied by a vivid conscious experience of the conceptual content such as in imagery [4]. Investigating unconscious automatic word processing though precludes a possible contribution of strategic imagery processes. Unconscious word processing can be probed by using a masked priming paradigm: Pattern masks (e.g., a random sequence of letters) which are displayed before and after a prime word stimulus [37], [38] eliminate conscious perception of prime words, although they still trigger cognitive processes at several levels of complexity including semantic processing (for reviews, see [37], [39], [40]–[43]). Unconscious word processing can be then measured via subliminal priming effects (e.g., facilitation) by masked prime words on subsequent processing of visible targets [44]–[47]. An earlier behavioral study found masked priming effects of spatial concepts in line with predictions of grounded cognition theory [38]. Combining the masked priming procedure with repetition priming of sound- and action-related concepts in a previous ERP study [48], we found differential repetition priming effects as a function of feature type: For action concepts, priming reduced a positive fronto-central ERP, whereas for sound concepts priming reduced a negative fronto-central ERP. This suggests that modality-specific conceptual information is accessed automatically even under unconscious viewing conditions. Similarly, Boulenger and colleagues [16] used pattern masks to investigate the effect of subliminally presented action verbs during movement preparation in a response priming paradigm. These verbs denoting actions performed with the hand/arm (e.g., write/throw) interfered with preparation and subsequent execution of an arm reaching movement showing that cortical structures that serve motor action are indeed part of action language processing thereby excluding any contribution of motor imagery.

However, these previous studies investigated unconscious repetition of identical action versus sound words or stimuli associated with similar action requirements (response congruency effect). In the present ERP study, we therefore use a novel feature-priming paradigm in order to test whether unconscious activation of a given feature dimension by a prime suffices to elicit priming on a target concept that exhibits a high relevance of the same feature dimension, but is otherwise not related to the prime with respect to global semantic association as well as to orthographic, phonological or response similarity. This feature-priming paradigm has the advantage over previous approaches to study (subliminal) conceptual feature-specific processing while such possibly confounding factors which may contribute to priming in repetition or response priming paradigms can be ruled out. Grounded cognition theory predicts that presenting a concept with high relevance of a given feature (e.g., action: typewriter or acoustic: banjo) as prime should pre-activate the corresponding sensory or motor brain region and facilitate subsequent processing of concepts with high relevance of the same feature type (e.g., inline skater or thunder, respectively) resulting in reduced sensory or motor activity. In support of our theoretical reasoning, behavioral costs (increased reaction times) emerged when participants had to switch between feature dimensions in property verification [49] suggesting that processing of concepts within one feature dimension is facilitated.

In our feature-priming paradigm, participants were visually presented with a masked prime word and a subsequent fully visible target word. Participants' task was to silently and attentively read the visible target words. In the feature-priming condition, both prime and target were action or sound words activating the same feature type (e.g., action: *typewriter – inline skater*; sound: *banjo – thunder*). Control conditions consisted either of action or sound target words, which were preceded by matched control words with low relevance for the corresponding feature type (non-priming 1; e.g., action: *street light – typewriter*; sound: *cradle – banjo*) or of action or sound prime words followed by control target words, respectively (non-priming 2; e.g., action: *inline skater – fir cone*, sound: *thunder – cord*). We used electrophysiological recordings of brain activity, because they are an ideal tool to capture fast decaying unconscious processes due to their high temporal resolution [50], [51].

**Figure 1:**
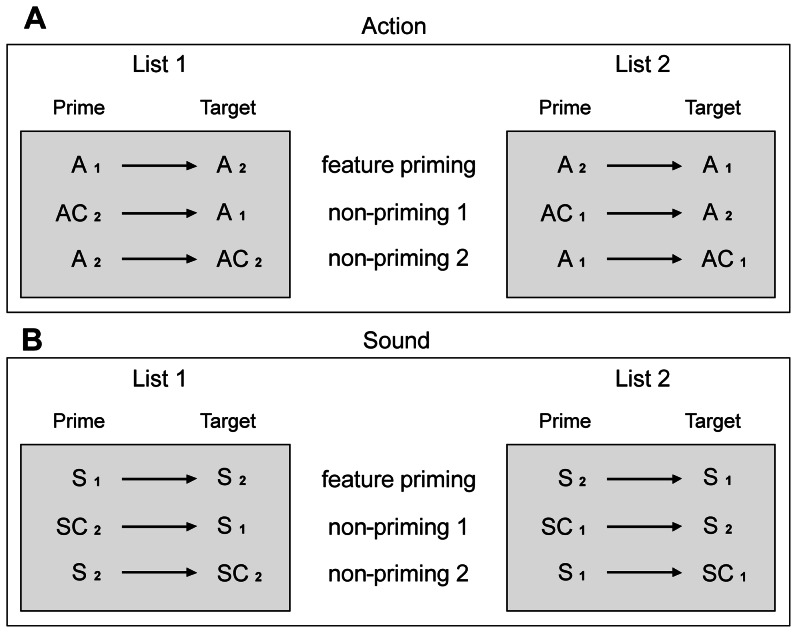
Stimulus lists with different prime target pairings for each condition. The two lists were counterbalanced across participants so that overall the same critical words of each feature type appeared in the feature- and in the non-priming conditions as targets. A_1_/A_2_: first/second set of critical action words, AC_1_/AC_2_: first/second set of action control words, S_1_/S_2_: first/second set of critical sound words, SC_1_/SC_2_ first/second set of sound control words.

The two control conditions allowed for differentiation between real priming and merely prime-related effects, which are elicited by prime processing itself and not by its impact on target processing: Real feature-priming effects are present only if ERPs of the priming condition differ from the first control condition (criterion 1 for priming). The first and second control conditions, in turn, should elicit similar ERPs (criterion 2 for priming). In contrast, an ERP effect can be considered as prime-related if ERPs of the priming condition differ from the first non-priming condition, but resemble those of the second non-priming condition. This is the case, because in both the priming and second non-priming condition primes were words of high feature relevance, whereas primes in the first non-priming condition consisted of control words (see Fig 1). The critical comparison therefore concerns the feature-priming versus first control conditions, whereas the second control conditions serve to discriminate between prime-related (non-priming 2 similar to feature-priming) and real priming effects (non-priming 2 similar to non-priming 1).

Previous studies showed that compared with control words action-related words elicited more positive ERPs at fronto-central electrodes [20], [21], [28], [52] whereas sound-related words evoked more negative ERPs over the very same scalp region [11]. Furthermore, modality-specific brain activation [11], [53], [54] and masked (repetition) priming effects [55], [56] already emerge between 100-200 ms after target stimulus onset. Subliminal ERP priming effects in the present study should therefore differ as a function of feature type over fronto-central scalp regions in the time interval of the N1: Priming of action features should specifically diminish the fronto-central positivity associated with action word processing, resulting in a relatively less positive potential. Priming of sound features, instead, should reduce the fronto-central negativity associated with sound word activations, resulting in a relatively more positive potential. Additionally, source analyses of scalp ERPs should reveal a differential activity pattern for sound and action priming close to the corresponding modality-specific cortex.

As predicted by grounded cognition theories of conceptual memory, such a differential pattern of ERP feature-priming effects evoked solely by subliminal pre-activation of the same feature dimension would substantially support a differential automatic activation of conceptual action and sound features independent of strategic imagery or semantic elaboration processes. This result pattern would be, however, difficult to reconcile with amodal theories.

## Materials and Methods

### Participants

22 (11 women) right-handed [57] native German-speaking volunteers (mean age = 21.8 years, ranging from 20 to 25 years) with normal or corrected-to normal visual acuity and without any history for neurological or psychiatric disorders participated in the ERP study.

### Ethics Statement

This study has been approved by the local Ethical Committee (permit number 217/07). All participants gave written informed consent to participate in this study. Subjects were paid for participating.

### Stimuli

320 words denoting objects with high (action-related) or low (action control) relevance of action features and high (sound-related) or low (sound control) relevance of acoustic features were used to create word pairs consisting of a prime and a target word for the feature-priming and the non-priming conditions. The average word length of primes and targets was 4.5 cm (7.6 characters), which resulted at a viewing distance of 75 cm in an average visual angle of 3.4°. Stimuli were drawn from an earlier study [48]: Two matched sets of action-related (n = 40 for each set) versus action control (n = 40 for each set) and sound-related (n = 40 for each set) versus sound control words (n = 40 for each set) were formed, which differed significantly only with regard to the relevance of the critical features (action-/sound-related vs. action/sound control p<.001), but were comparable for the relevance of visual features, familiarity and emotion (all p>.05; see Table 1). Sets were also matched for word length (all p>.5) and word frequency (all p>.5 according to the CELEX lexical data base [58]) and they were balanced for the number of words denoting natural (e.g. animals) versus artifact (e.g., tools) objects. Control words and critical words thus cover the same range of meanings or semantic categories. When action and sound words were directly compared significant differences between the critical feature dimension were observed in both subsets (all p<.001). It should be noted however that sound words showed a relatively high association with non-critical action features presumably reflecting the fact that sound is frequently produced by specific actions with objects (e.g. ringing a bell), whereas action words exhibited a relatively low association with the non-critical acoustic features.

**Table 1:** Conceptual and linguistic variables for the critical word sets referring to action- and sound-related concepts and the corresponding control word sets as well as p-values of two-tailed t-tests.

	action	acoustic	visual	familiarity	emotion	word length	word frequency
1^st^ set							
action-related	4.40	1.74	4.20	4.06	2.61	7.65	29.83
action control	1.77	1.59	4.24	3.89	2.45	7.55	32.93
p-value	<.001	.51	.79	.32	.33	.87	.90
sound-related	3.25	5.13	3.94	3.69	2.80	7.70	29.88
sound control	3.30	1.11	3.89	3.86	2.57	7.50	28.80
p-value	.79	<.001	.78	.46	.21	.80	.96
2^nd^ set							
action-related	4.39	1.78	4.16	4.06	2.61	7.68	30.00
action control	1.77	1.61	4.29	3.77	2.54	7.55	33.98
p-value	<.001	.44	.40	.11	.69	.83	.80
sound-related	3.24	5.19	3.73	3.54	2.60	7.65	29.48
sound control	3.30	1.13	4.07	3.88	2.52	7.50	28.35
p-value	.71	<.001	.09	.08	.65	.83	.92

Variables where measured on a scale from 1 (no associations) to 6 (strong associations) by asking participants in a norming study how strong they associate a named object with actions they can perform with it, how strong they think of typical sounds the object produces, how strong they associate visual features like contour or color with the object, how they associate emotions with the object and how familiar the object is for them.

In the two feature-priming conditions (action, sound) both primes and targets were critical words from the sets of action-related and sound-related words, respectively: action priming (e.g., *typewriter – inline skater*; n = 40 pairs) and sound priming (e.g., *banjo – thunder*; n = 40 pairs). In the first non-priming conditions, the target word was either an action or a sound word, whereas the prime word was a corresponding control word: action non-priming 1 (e.g., *street light – typewriter*; n = 40 pairs) and sound non-priming 1 (e.g., *cradle – banjo*; n = 40 pairs). Two stimulus lists (see Material S1) with different prime target pairings were created such that the targets of the feature-priming and the first non-priming conditions of each conceptual feature type were exchanged in these lists in order to avoid repetition of the same targets (see Fig. 1): In one list, words with high relevance of a given feature type served as targets in the feature-priming condition and in the other list they served as targets in the first non-priming condition and vice versa. Stimulus lists were counterbalanced across participants so that overall the same words of each feature type appeared as targets in the feature- and in the first non-priming conditions.

In the second non-priming conditions, primes were words with high feature relevance identical to the target words of the feature-priming condition, but the targets were corresponding control words identical to the prime words of the first non-priming condition: action non-priming 2 (e.g., *inline skater – fir cone*; n = 40 pairs) and sound non-priming 2 (e.g., *thunder – cord*; n = 40 pairs). So, within one list every word appears twice: once as a target and once as a prime (Fig. 1).

The second non-priming condition not only equilibrated the stimulus material in terms of presentation frequency of masked prime words as visible targets, but also allowed for differentiation between real priming and prime-related effects (see introduction). When creating the word pairs for the two lists, the length of prime and target words in each trial was equated as much as possible, and it was ensured that prime and target did not show global semantic or orthographic similarity.

### Procedure

#### Silent reading task

**Figure 2:**
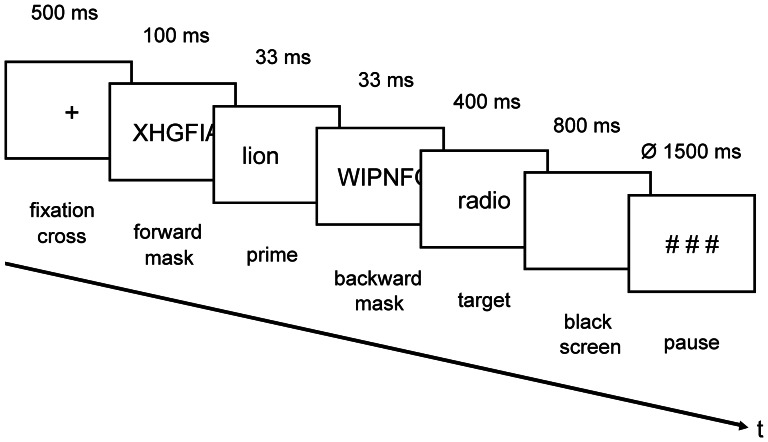
Experimental procedure of the masked feature-priming task.

ERP recordings were performed in a dimly lit sound-attenuated and electrically shielded booth. Participants were seated upright in front of a computer screen at a viewing distance of 75 cm. Participants were instructed to remain relaxed and restrict their blinks and eye movements to the pauses between trials. The word pairs were visually presented on a computer screen in white font on a black background synchronously with the screen refresh (16.67 ms). Each of the 240 trials, subdivided into six blocks of 40 trials, started with the presentation of a fixation cross (500 ms; see Fig. 2) followed by a forward mask (100 ms), a prime word (33 ms) and a backward mask (33 ms). Each mask consisted of 14 randomly selected capital letters. Immediately after the backward mask, a target word was shown for 400 ms and the screen went black for 800 ms. Prime and target were written in lower case except for their first letter, which was a capital letter (see Material S1). At last, three hash marks indicated a break between trials lasting at an average of 1500 ms (varying randomly from 1000 to 2000 ms). Participants were asked to read the target words silently and attentively. This silent reading task, which did not require an overt response, was administered in order to avoid contamination of ERPs by motor activity.

#### Masked prime identification test\

Directly after the main experiment, participants were informed of the prime words shown between the masks and were asked whether they had recognized these. None of the participants reported awareness of the primes. For a more objective measurement of prime recognition, a masked prime identification test was administered [50] . Participants performed a simple visual discrimination task on masked stimuli consisting of 40 words and 40 letter strings. Each letter string comprised 7 repetitions of the identical capital letter (e.g., DDDDDDD), which was randomly selected in each trial. Trial sequence in this task was the same as in the silent reading task. Participants' task was to decide whether the masked stimulus was a word or a letter string. Instructions stressed accuracy over response speed. Participants were also requested to make the best guess when they did not feel confident about the correct response. Participants' indicated their response by button presses with the index (word) and the middle finger (letter string), respectively. This prime identification test depends on simple visual discrimination of stimulus features and is therefore highly sensitive with respect to residual conscious vision [50].

### ERP recordings, signal extraction and data analysis

Scalp potentials were recorded using an equidistant montage of 64 sintered Ag/AgCl electrodes mounted in an elastic cap (EasyCap, Munich, Germany). An additional electrode between FPz and Fz was connected to the ground and another one between Cz and FCz was used as recording reference. Eye movements and blinks were recorded with 4 (out of the 64) electrodes placed beneath and laterally to the eyes. Impedances of all electrodes were kept below 5 kΩ. Electroencephalography (EEG) signals were amplified with Brainamp amplifiers (BrainProducts, Gilching, Germany; low-pass filter: 70 Hz, 24 dB/octave attenuation; 50 Hz notch filter) and continuously recorded with a digitalization rate of 500 Hz. Using the BrainVisionAnalyzer software (BrainProducts, Gilching, Germany) electrical signals were digitally bandpass filtered (low cutoff: 0.1 Hz, 12 dB/octave; high cutoff: 30 Hz, 24 dB/octave) and corrected for ocular artifacts using independent component analysis [59]. Continuous EEG was segmented in epochs starting -320 ms before target stimulus onset to allow for a 153 ms baseline correction prior to the onset of the forward mask (at −167 ms) and ending 1000 ms after target stimulus onset. Baseline correction was performed prior to the onset of the forward mask in order to avoid distortion of the baseline by visually evoked potentials to the mask. Thereafter, artifact-free EEG segments were averaged separately for each of the four experimental conditions. Like in the most relevant previous studies [11], [20], [52], [60], with which we want to compare our data, an average-reference transformation [61], [62] was performed to obtain reference independent estimations of scalp voltages.

Statistical analysis focused on a fronto-central scalp region of interest, where action- as well as sound-related potentials are typically recorded [11], [20], [21], [28], [63] including three pairs of contralateral electrodes: AF3/AF4, F1/F2 and FC1/FC2. ERPs of the six experimental conditions mainly differed in the N1 (100–180 ms) time interval [11], [53]–[56]. Mean voltages within this time window were calculated and subjected to a repeated-measures analyses of variance (ANOVAs) with the factors feature type (action vs. sound), priming (feature-priming vs. non-priming 1 vs. non-priming 2), hemisphere (left vs. right) and electrode site. An additional ANOVA in the time interval between -320 ms and target word onset (at 0 ms) was performed to exclude that any pre-target activity might have compromised possible priming effects. This analysis did not reveal any significant effects (all p>.10).

Neural sources for significant ERP feature-priming effects were determined using distributed source modeling based on minimum norm source estimates [64] implemented in BESA 5.1 (MEGIS). Sources were computed for the grand-average ERP difference waves between the action/sound feature-priming and the first action/sound non-priming conditions, respectively. Minimum norm source estimates were calculated using a standardized realistic finite element head model (FEM). The pre-target baseline was used to estimate the noise regularization parameters. Minimum norm was computed with depth weighting, spatio-temporal weighting and noise weighting for individual channels, the default parameters of BESA, which were also applied in our previous work [11], [52], [65]. Depth weighting reduces source localization bias towards superficial currents due to an attenuation of EEG lead fields with increasing source depth [66], [67]. Spatio-temporal weighting gives larger weight to sources more likely assumed to contribute to the recorded data based on the signal subspace correlation measure [68]. Brodmann areas (BA) of peak activity were estimated using the Talairach Daemon [69].

## Results

### Masked prime identification

Mean accuracy in the masked prime identification test was 51.5% and did not significantly deviate from the chance level of 50% (t(21) = 1.49, p = .15), which is expected when merely guessing. *D*' sensitivity measures [70] calculated from participants' hit rates (correct responses to words) and false alarm rates (incorrect responses to capital letter strings) did not significantly deviate from zero (*d*′ = .06, t(21) = 1.05, p = .31). D' separately calculated for action and sound words also did not significantly deviate from zero (action: *d′* = −0.03, t(21) = −0.21, p = .84; sound: *d′* = .14, t(21) = 1.24, p = .23). Hence, participants could not consciously distinguish between words and letter strings in either condition.

### Electrophysiological results

**Figure 3:**
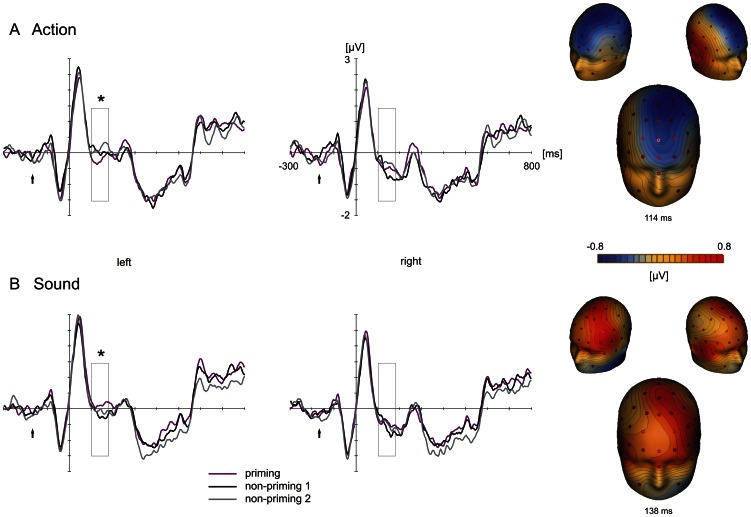
Grand-average ERPs from the fronto-central scalp region of interest as a function of feature type and prime modality (feature-priming vs. non-priming 1 vs. non-priming 2). Shown are the average ERP waveforms elicited by action-related (A) and sound-related (B) words as well as topographic maps of the corresponding action and sound priming effects (difference wave of feature-priming vs. non-priming 1) recorded at its maximum global field power. Black rectangles highlight the critical time window (100-180 ms). Significant priming effects are indicated with a black asterisk and little black arrows denote the onset of the forward mask followed by the prime word and the backward mask. The y-axes indicates the onset of the target word.

Starting at about 100 ms after stimulus onset, unprimed action words (non-priming 1 condition) elicited a relatively more positive, sound words a relatively more negative scalp potential at fronto-central electrodes (see Fig. 3). A comparison of the feature-priming with the first non-priming conditions (unprimed words of high feature relevance) revealed that priming of action features led to a negative potential shift, whereas priming of sound features lead to a positive potential shift. ERPs of the priming conditions differed from the first non-priming conditions, which, in turn, overlapped with the second non-priming condition. This ERP pattern indicates that the obtained differences reflect real priming effects and not prime-related activity (see introduction). ERP priming effects were statistically analyzed in the time interval of the N1 (100-180 ms) over the fronto-central scalp (for the rationale of time window and electrode site selection, see the methods section).

### N1 time interval (100-180 ms)

A repeated-measures analysis of variance (ANOVA) with the factors feature type, priming, hemisphere and electrode site revealed a significant interaction between feature type, priming and hemisphere (F_(2,42)_ = 4.29; p = .02). Post-hoc Newman-Keuls tests further qualified this interaction: For action words, statistically significant potential differences were found between the feature-priming and the first non-priming condition (p<.05), confirming priming criterion 1, as well as between the feature-priming and the second corresponding non-priming conditions (p<.01) over the left hemisphere. Primed words elicited a more negative scalp potential than non-primed ones. The two non-priming conditions in turn did not differ from each other (p>.69) confirming priming criterion 2. For sound words, the feature-priming condition also significantly differed from the corresponding first non-priming condition (p<.03; confirming criterion 1) and showed a trend for a difference to the second non-priming condition (p<.1) over the left hemisphere. Here, the primed sound words evoked a more positive scalp potential than the non-primed ones. In line with criterion 2, the two non-priming conditions again did not differ from each other (p>.42). Thus, over the left hemisphere, feature-priming effects for action words exhibited the opposite polarity of the effects for sound words. Furthermore, comparing ERPs of the non-primed action with the non-primed sound words revealed a statistically significant difference over the left hemisphere (p<.05): Non-primed action words elicited a more positive scalp potential than non-primed sound words. No significant effects were found over the right hemisphere. (Fig. 3)

**Figure 4:**
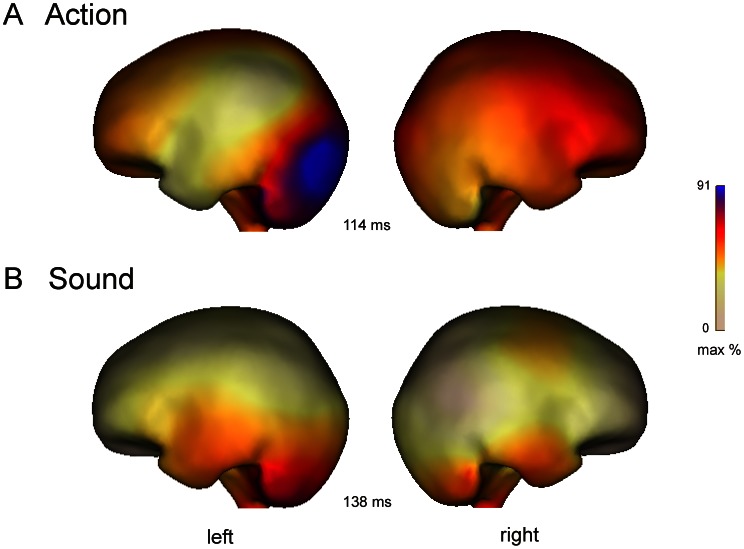
Neural source estimates of scalp potentials calculated according to the minimum norm algorithm from ERP difference waves as a function of feature type. (A) Source of the significant action feature-priming effect (B) Source of the significant sound feature-priming effect.

Source analysis of the priming effects in the action condition indicated activity in right frontal cortex in and close to BA 6 extending into right parietal (BA 7) cortex as well as in BA 9 and10 and in left occipital brain areas (BA 18, 19, see Fig. 4A). Source analysis of feature-priming effects for sound words also suggested neural generators in left occipital brain areas (BA 18,19), but revealed additional activity in bilateral temporal (BA 20, 21 and 38) and frontal (BA 6) cortex (Fig. 4B).

## Discussion

In the present ERP study we investigated for the first time unconscious automatic processing of action- and sound-related concepts within a masked feature-priming paradigm. This paradigm tested the prediction of grounded cognition theory whether pre-activation of the conceptual action versus sound feature dimension by an unconscious prime word differentially modulates subsequent processing of a target word denoting a concept with high relevance of the same feature dimension, respectively. In line with our predictions, we found feature-specific subliminal priming effects at left-hemispheric fronto-central electrode sites between 100 and 180 ms post target stimulus onset differing with regard to topography (polarity differences) and neural sources: Compared to the respective non-priming conditions, feature-priming in action words was associated with relatively more negative ERPs, whereas feature-priming in sound words elicited relatively more positive ERPs. Source analyses suggested feature-specific neural generators in or close to modality-specific areas. In line with grounded cognition theory, these differential subliminal feature-priming effects indicate rapid automatic access to conceptual action versus sound features also under unconscious viewing conditions thereby excluding any post-conceptual strategic processes.

Consistent with earlier ERP studies with visible stimuli, non-primed action words evoked a relatively more positive scalp potential at fronto-central electrodes [20], [21], [28], [52]. Non-primed sound words, in contrast, evoked a relatively more negative potential at the very same scalp location [11], [48]. Based on that, we now found in the present study feature-specific masked priming effects: Compared to the non-primed action words (first non-priming condition), the positivity to action words was specifically reduced in the priming condition; ERPs of primed sound words, in contrast, resulted in a reduction of the relatively negative brain potential of non-primed sound words. As ERPs of the action and sound priming condition differed from the corresponding first non-priming conditions and the respective first and second non-priming conditions were similar to each other, we can claim that the obtained ERP effects are real priming effects and do not represent prime-related activity.

Furthermore, all stimuli used in the masked feature-priming paradigm were the same in the priming and the first non-priming condition across participants. Therefore, these feature-specific subliminal priming effects cannot be influenced by non-semantic lexical effects due to possible unnoticed insufficient stimulus matching. As both critical and control words were presented as targets, the likelihood for repetition of a prime as a target word (and vice versa) from a preceding to a subsequent trial within one participant was the same for the experimental and the two control conditions. Thus, priming effects rather must arise from differential unconscious access to action- versus sound-related conceptual features of the primes thereby pre-activating the same feature-dimension of the subsequently presented corresponding visible target words.

Source analyses of the ERP effects suggested partially different neural generators for the action and sound feature-priming effects: Source activity specific for action priming was revealed in right frontal and parietal cortex whereas source activity specific for sound priming was obtained in bilateral temporal and frontal areas. The observed feature-specific activity is compatible with previous findings using visible stimulation, which suggest a representation of action features in frontal and parietal areas close to premotor cortex [52], [60], [71] and a representation of sound features in temporal areas close to auditory association cortex [11]. Additional source activity of sound priming effects in frontal cortex is presumably based on the fact that unconscious processing of sound words activates associated actions in frontal motor areas that typically produce the corresponding sound (see also the relatively strong association of sound words with action features, Table 1). Regarding laterality differences between source localization of action and sound feature-priming effects, it should be noted that laterality of conceptual processing is variable to some extent: Activation differences were sometimes seen only in the language dominant left hemisphere and sometimes across hemispheres [72] or even more pronounced in the right (non-dominant) hemisphere during processing of action information [24], [31], [73]–[75]. At present, the factors determining the precise hemispheric lateralization are not fully understood. Source activity in left occipital areas though was found for both action and sound feature-priming effects possibly reflecting top-down influences from activated feature-specific representations on the processing of the visual word form (see for example [76].) Our source analyses, though, are only descriptive so that the obtained neural generators have to be interpreted with caution. It also might be informative to additionally perform a localizer experiment, e.g. with simple tones and finger movements, for a more precise mapping of modality-specific acoustic and motor regions.

The subliminal feature-specific priming effects in this study provide important evidence for grounded cognition theory, which predicts differential processing of action and sound words in corresponding modality-specific brain systems: Firstly, polarity as well as topography of the scalp potentials to the unprimed action and sound word conditions, perfectly agree with previous ERP findings of visible action and sound word processing that were related to differential activation in motor and auditory cortex [11], [77]. Secondly, subliminal feature-priming was associated with a distinct reduction of this feature-specific activity resulting in differential feature-priming effects. Hence, these topographic differences in subliminal feature-priming suggest that action and sound words are unconsciously processed in different brain areas, presumably in corresponding modality-specific cortex. Thirdly, although the present source analyses are only descriptive and therefore have to be interpreted with caution, they reveal differential neural generators for the action and sound ERP feature-priming effects in or close to motor and auditory areas, respectively. This pattern of subliminal feature-specific priming effects is difficult to reconcile with amodal theories, which do not assume a neuroanatomical distinct representation of conceptual information as a function of feature type.

As ERP recordings provide only correlational information, we cannot demonstrate the causal relevance of modality-specific cortex for subliminal feature-specific processing, in order to satisfy a further important prediction of grounded cognition theory [4], [78]. However, at least for visible processing of action and sound words, an intact motor [24] or auditory cortex [18] has been shown to be necessary for appreciating the meaning of the corresponding word category, indicating the causal relevance of modality-specific cortex for conceptual processing. Nevertheless, it would be very interesting to explore our subliminal feature-priming paradigm in combination with transcranial magnetic stimulation (TMS) to test the causality of motor and auditory cortex for the emergence of subliminal feature-specific priming effects.

The functional relevance of modality-specific information for conceptual processing is also suggested by a previous behavioral study investigating modality-specific switching costs [49]. Results demonstrate that processing of conceptual information within one feature dimension facilitates corresponding property verifications. Here, we extend these findings by demonstrating that feature-priming effects can also be observed for subliminally presented masked primes. As masked priming effects reflect unconscious automatic processing [37], [79] feature-specific conceptual processing cannot be influenced by post-conceptual strategies like visual imagery or semantic elaboration (cf., [35], [80]). The present results therefore confirm the assumption of grounded cognition theory that activity in sensory and motor areas during conceptual processing can also occur unconsciously and is not mandatorily accompanied by a vivid conscious experience of the conceptual content such as in imagery [4]. However, our findings do not exclude the possibility of a supramodal “conceptual hub” - a convergence zone [81] located in anterior temporal cortex, which integrates distributed conceptual representations in the sensory and motor brain areas into a coherent concept as suggested previously [3], [65], [82]–[84].

In conclusion, the present observation of specific feature-priming effects, solely elicited by subliminal pre-activation of the same action or sound feature dimension instead of identical or congruent presentation of action or sound words, respectively, demonstrates that processing of conceptual action and sound features automatically activate separable brain circuits presumably in or close to corresponding modality-specific cortex. As feature-specific processing occurs under unconscious viewing conditions, post-conceptual strategic processes such as imagery or semantic elaboration can be excluded. Taken together, the present results confirm and extend previous studies suggesting a grounding of concepts in perception and action and provide further important evidence for a modality-specific organization of conceptual memory.

## Supporting Information

Material S1 Stimulus Lists.
(DOC)
