# Supplementary material for: Masked Priming of Conceptual Features Reveals Differential Brain Activation during Unconscious Access to Conceptual Action and Sound Information
Source: PLoS One. 2013 May 31;8(5):e65910. doi: 10.1371/journal.pone.0065910 (PMC3669239; doi:10.1371/journal.pone.0065910)
Supplement: Material S1 — Stimulus Lists. (DOC) [file pone.0065910.s001.doc]

**S1: Stimulus Lists**

**List 1 of the action priming condition (cf. A_1_ → A_2_, Fig. 1)**

| **German stimulus list** | | **English translation** | |
| --- | --- | --- | --- |
| **prime** | **target** | **prime** | **target** |
| Schraubenzieher | Johannisbeere | screwdriver | currant |
| Schreibmaschine | Inline-Skater | typewriter | inline skater |
| Fleischklopfer | Schreibfeder | meat tenderizer | quill |
| Schnittlauch | Korkenzieher | chives | corkscrew |
| Staubsauger | Essstäbchen | vacuum cleaner | chopstick |
| Kochlöffel | Clementine | cooking spoon | clementine |
| Malerwalze | Küchenwaage | paint roller | kitchen scales |
| Wagenheber | Haselnuss | jack | hazelnut |
| Aubergine | Hammer | aubergine | hammer |
| Blaubeere | Regenschirm | blueberry | umbrella |
| Brombeere | Tastatur | blackberry | keyboard |
| Kokosnuss | Heckenschere | coconut | hedge clippers |
| Kopfsalat | Frisbee | lettuce | frisbee |
| Mikroskop | Gießkanne | microscope | watering can |
| Bumerang | Zwetschge | boomerang | plum |
| Getreide | Rollschuhe | cereal | rollerblades |
| Himbeere | Messer | raspberry | knife |
| Peitsche | Garten | whip | garden |
| Pfirsich | Zither | peach | zither |
| Webstuhl | Feldsalat | loom | lamb's lettuce |
| Fahrrad | Spargel | bicycle | asparagus |
| Kirsche | Surfbrett | cherry | surfboard |
| Paprika | Gameboy | pepper | gameboy |
| Zitrone | Pfad | lemon | path |
| Ananas | Pferd | pineapple | horse |
| Banane | Spaten | banana | spade |
| Löffel | Setzling | spoon | seedling |
| Pinsel | Aprikose | brush | apricot |
| Tandem | Peperoni | tandem | chillies |
| Birne | Liane | pear | liana |
| Handy | Zwiebel | cellphone | onion |
| Lauch | Kajak | leek | kayak |
| Mango | Lupe | mango | magnifier |
| Olive | Axt | olive | axe |
| Zange | Tomate | pliers | tomato |
| Beil | Kiwi | hatchet | kiwi |
| Herd | Melone | cooker | melon |
| Kamm | Apfel | comb | apple |
| Reis | Hobel | rice | plane |
| Bob | Gurke | bobsled | cucumber |

**List 1 of the first action non-priming condition (cf. AC_2_ → A_1_, Fig. 1)**

| **German stimulus list** | | **English translation** | |
| --- | --- | --- | --- |
| **prime** | **target** | **prime** | **target** |
| Tannenzapfen | Schraubenzieher | fir cone | screwdriver |
| Straßenlampe | Schreibmaschine | street light | typewriter |
| Sockelleiste | Fleischklopfer | baseboard | meat tenderizer |
| Tintenfisch | Schnittlauch | squid | chives |
| Osterglocke | Staubsauger | daffodil | vacuum cleaner |
| Wetterhahn | Kochlöffel | weathercock | cooking spoon |
| Leitplanke | Malerwalze | guardrail | paint roller |
| Fenstersims | Wagenheber | window ledge | jack |
| Windspiel | Aubergine | wind chimes | aubergine |
| Dachziegel | Blaubeere | tile | blueberry |
| Stimmband | Brombeere | vocal chord | blackberry |
| Grabstein | Kokosnuss | tombstone | coconut |
| Skulptur | Kopfsalat | sculpture | lettuce |
| Fundament | Mikroskop | base | microscope |
| Schnecke | Bumerang | snail | boomerang |
| Schatten | Getreide | shade | cereal |
| Abbildung | Himbeere | illustration | raspberry |
| Vogelbeere | Peitsche | rowan berry | whip |
| Schwalbe | Pfirsich | swallow | peach |
| Eidechse | Webstuhl | lizard | loom |
| Satellit | Fahrrad | satellite | bicycle |
| Denkmal | Kirsche | monument | cherry |
| Libelle | Paprika | dragonfly | pepper |
| Plakette | Zitrone | sticker | lemon |
| Meise | Ananas | tit | pineapple |
| Plakat | Banane | poster | banana |
| Käfer | Löffel | beetle | spoon |
| Seerose | Pinsel | water lily | brush |
| Gemälde | Tandem | painting | tandem |
| Fuge | Birne | gap | pear |
| Tanne | Handy | fir | cellphone |
| Beleg | Lauch | receipt | leek |
| Wurm | Mango | worm | mango |
| Säule | Olive | column | olive |
| Tulpe | Zange | tulip | pliers |
| Igel | Beil | hedgehog | hatchet |
| Wolf | Herd | wolf | cooker |
| Raupe | Kamm | caterpillar | comb |
| Star | Reis | starling | rice |
| Wal | Bob | whale | bobsled |

**List 1 of the second action non-priming condition (cf. A_2_ → AC_2_, Fig. 1)**

| **German stimulus list** | | **English translation** | |
| --- | --- | --- | --- |
| **prime** | **target** | **prime** | **target** |
| Johannisbeere | Sockelleiste | currant | baseboard |
| Inline-Skater | Tannenzapfen | inline skater | fir cone |
| Schreibfeder | Wetterhahn | quill | weathercock |
| Korkenzieher | Straßenlampe | corkscrew | street light |
| Essstäbchen | Dachziegel | chopstick | tile |
| Clementine | Abbildung | clementine | illustration |
| Küchenwaage | Leitplanke | kitchen scales | guardrail |
| Haselnuss | Stimmband | hazelnut | vocal chord |
| Hammer | Seerose | hammer | water lily |
| Regenschirm | Fundament | umbrella | base |
| Tastatur | Vogelbeere | keyboard | rowan berry |
| Heckenschere | Tintenfisch | hedge clippers | squid |
| Frisbee | Plakette | frisbee | sticker |
| Gießkanne | Windspiel | watering can | wind chimes |
| Zwetschge | Eidechse | plum | lizard |
| Rollschuhe | Fenstersims | rollerblades | window ledge |
| Messer | Schnecke | knife | snail |
| Garten | Plakat | garden | poster |
| Zither | Tulpe | zither | tulip |
| Feldsalat | Grabstein | lamb's lettuce | tombstone |
| Spargel | Denkmal | asparagus | monument |
| Surfbrett | Osterglocke | surfboard | daffodil |
| Gameboy | Libelle | gameboy | dragonfly |
| Pfad | Wal | path | whale |
| Pferd | Star | horse | starling |
| Spaten | Gemälde | spade | painting |
| Setzling | Skulptur | seedling | sculpture |
| Aprikose | Satellit | apricot | satellite |
| Peperoni | Schwalbe | chillies | swallow |
| Liane | Fuge | liana | gap |
| Zwiebel | Schatten | onion | shade |
| Kajak | Wurm | kayak | worm |
| Lupe | Tanne | magnifier | fir |
| Axt | Meise | axe | tit |
| Tomate | Igel | tomato | hedgehog |
| Kiwi | Raupe | kiwi | caterpillar |
| Melone | Beleg | melon | receipt |
| Apfel | Wolf | apple | wolf |
| Hobel | Käfer | plane | beetle |
| Gurke | Säule | cucumber | column |

**List 1 of the sound priming condition (cf. S_1_ → S_2_, Fig. 1)**

| **German stimulus list** | | **English translation** | |
| --- | --- | --- | --- |
| **prime** | **target** | **prime** | **target** |
| Klapperschlange | Lautsprecher | rattlesnake | loudspeaker |
| Bohnermaschine | Alarmanlage | floor polisher | alarm system |
| Küchenmaschine | Mundharmonika | kitchen machine | harmonika |
| Akkuschrauber | Trillerpfeife | cordless screwdriver | whistle |
| Kanarienvogel | Synthesizer | canary | synthesizer |
| Bohrmaschine | Dudelsack | drill | bagpipes |
| Kuckucksuhr | Hubschrauber | cuckoo clock | helicopter |
| Martinshorn | Wasserfall | siren (emergency vehicle) | waterfall |
| Kettensäge | Wellensittich | chainsaw | budgerigar |
| Klospülung | Kontrabass | flush | double bass |
| Nachtigall | Metronom | nichtingale | metronome |
| Eisenbahn | Rotkehlchen | railway | robin |
| Kopfhörer | Schwein | headphones | pig |
| Mandoline | Ventilator | mandolin | fan |
| Gewitter | Akkordeon | thunderstorm | accordion |
| Jagdhorn | Piepser | hunting horn | bleeper |
| Keyboard | Elefant | keyboard (musical instrument) | elephant |
| Saxophon | Türklingel | saxophone | doorbell |
| Triangel | Kuckuck | triangle | cuckoo |
| Klingel | Schnake | bell | crane fly |
| Papagei | Alphorn | parrot | alphorn |
| Violine | Schaf | violin | sheep |
| Glocke | Biene | bell | bee |
| Grille | Posaune | cricket | trombone |
| Hummel | Panflöte | bumblebee | panpipe |
| Specht | Megaphon | woodpecker | megaphone |
| Wecker | Frosch | alarm clock | frog |
| Banjo | Donner | banjo | thunder |
| Geige | Mofa | violin | moped |
| Mücke | Echo | mosquito | echo |
| Orgel | Hahn | organ | rooster |
| Radio | Löwe | radio | lion |
| Ziege | Cembalo | goat | cembalo |
| Eule | Sirene | owl | siren |
| Huhn | Pfeife | hen | pipe |
| Hund | Oboe | dog | oboe |
| Hupe | Cello | horn | cello |
| Rabe | Pauke | raven | kettledrum |
| Tuba | Krähe | tuba | crow |
| Uhu | Fön | eagle owl | hairdryer |

**List 1 of the first sound non-priming condition (cf. SC_2_ → S_1_, Fig. 1)**

| **German stimulus list** | | **English translation** | |
| --- | --- | --- | --- |
| **prime** | **target** | **prime** | **target** |
| Verkehrsschild | Klapperschlange | road sign | rattlesnake |
| Rosenkranz | Bohnermaschine | rosary | floor polisher |
| Kaktusfeige | Küchenmaschine | prickly pear | kitchen machine |
| Sonnenschirm | Akkuschrauber | sunshade | cordless screwdriver |
| Blumenkohl | Kanarienvogel | cauliflower | canary |
| Grapefruit | Bohrmaschine | grapefruit | drill |
| Zuckerrohr | Kuckucksuhr | sugar cane | cuckoo clock |
| Betonkübel | Martinshorn | concrete bucket | siren (emergency vehicle) |
| Sitzkissen | Kettensäge | cushion | chainsaw |
| Reisepass | Klospülung | passport | flush |
| Lattenrost | Nachtigall | slatted frame | nichtingale |
| Garderobe | Eisenbahn | coatrack | railway |
| Steinpilz | Kopfhörer | cep | headphones |
| Kakerlake | Mandoline | cockroach | mandolin |
| Sattel | Gewitter | saddle | thunderstorm |
| Zucchini | Jagdhorn | zucchini | hunting horn |
| Broccoli | Keyboard | broccoli | keyboard (musical instrument) |
| Gasmaske | Saxophon | gas mask | saxophone |
| Holzkiste | Triangel | wooden box | triangle |
| Balkon | Klingel | balcony | bell |
| Kalender | Papagei | calendar | parrot |
| Sessel | Violine | armchair | violin |
| Weinrebe | Glocke | grapevine | bell |
| Bohne | Grille | bean | cricket |
| Sichel | Hummel | sickle | bumblebee |
| Papaya | Specht | papaya | woodpecker |
| Avocado | Wecker | avocado | alarm clock |
| Wiege | Banjo | cradle | banjo |
| Hummer | Geige | lobster | violin |
| Hocker | Mücke | stool | mosquito |
| Hut | Orgel | hat | organ |
| Spiegel | Radio | mirror | radio |
| Korb | Ziege | basket | goat |
| Linse | Eule | lens | owl |
| Mütze | Huhn | cap | hen |
| Draht | Hund | wire | dog |
| Schnur | Hupe | cord | horn |
| Kanne | Rabe | pot | raven |
| Sesam | Tuba | sesame | tuba |
| Pult | Uhu | lectern | eagle owl |

**List 1 of the second sound non-priming condition (cf. S_2_ → SC_2_, Fig. 1)**

| **German stimulus list** | | **English translation** | |
| --- | --- | --- | --- |
| **prime** | **target** | **prime** | **target** |
| Lautsprecher | Blumenkohl | loudspeaker | cauliflower |
| Alarmanlage | Rosenkranz | alarm system | rosary |
| Mundharmonika | Kaktusfeige | harmonika | prickly pear |
| Trillerpfeife | Grapefruit | whistle | grapefruit |
| Synthesizer | Zuckerrohr | synthesizer | sugar cane |
| Dudelsack | Zucchini | bagpipes | zucchini |
| Hubschrauber | Sonnenschirm | helicopter | sunshade |
| Wasserfall | Garderobe | waterfall | coatrack |
| Wellensittich | Verkehrsschild | budgerigar | road sign |
| Kontrabass | Betonkübel | double bass | concrete bucket |
| Metronom | Gasmaske | metronome | gas mask |
| Rotkehlchen | Sitzkissen | robin | cushion |
| Schwein | Sessel | pig | armchair |
| Ventilator | Reisepass | fan | passport |
| Akkordeon | Lattenrost | accordion | slatted frame |
| Piepser | Sichel | bleeper | sickle |
| Elefant | Holzkiste | elephant | wooden box |
| Türklingel | Steinpilz | doorbell | cep |
| Kuckuck | Papaya | cuckoo | papaya |
| Schnake | Weinrebe | crane fly | grapevine |
| Alphorn | Bohne | alphorn | bean |
| Schaf | Balkon | sheep | balcony |
| Biene | Mütze | bee | cap |
| Posaune | Avocado | trombone | avocado |
| Panflöte | Broccoli | panpipe | broccoli |
| Megaphon | Kakerlake | megaphone | cockroach |
| Frosch | Hocker | frog | stool |
| Donner | Schnur | thunder | cord |
| Mofa | Sesam | moped | sesame |
| Echo | Korb | echo | basket |
| Hahn | Wiege | rooster | cradle |
| Löwe | Spiegel | lion | mirror |
| Cembalo | Draht | cembalo | wire |
| Sirene | Sattel | siren | saddle |
| Pfeife | Kalender | pipe | calendar |
| Oboe | Hut | oboe | hat |
| Cello | Kanne | cello | pot |
| Pauke | Hummer | kettledrum | lobster |
| Krähe | Linse | crow | lens |
| Fön | Pult | hairdryer | lectern |

**List 2 of the action priming condition (cf. A_2_ → A_1_, Fig. 1)**

| **German stimulus list** | | **English translation** | |
| --- | --- | --- | --- |
| **prime** | **target** | **prime** | **target** |
| Johannisbeere | Schraubenzieher | currant | screwdriver |
| Inline-Skater | Schreibmaschine | inline skater | typewriter |
| Schreibfeder | Fleischklopfer | quill | meat tenderizer |
| Korkenzieher | Schnittlauch | corkscrew | chives |
| Essstäbchen | Staubsauger | chopstick | vacuum cleaner |
| Clementine | Kochlöffel | clementine | cooking spoon |
| Küchenwaage | Malerwalze | kitchen scales | paint roller |
| Haselnuss | Wagenheber | hazelnut | jack |
| Hammer | Aubergine | hammer | aubergine |
| Regenschirm | Blaubeere | umbrella | blueberry |
| Tastatur | Brombeere | keyboard | blackberry |
| Heckenschere | Kokosnuss | hedge clippers | coconut |
| Frisbee | Kopfsalat | frisbee | lettuce |
| Gießkanne | Mikroskop | watering can | microscope |
| Zwetschge | Bumerang | plum | boomerang |
| Rollschuhe | Getreide | rollerblades | cereal |
| Messer | Himbeere | knife | raspberry |
| Garten | Peitsche | garden | whip |
| Zither | Pfirsich | zither | peach |
| Feldsalat | Webstuhl | lamb's lettuce | loom |
| Spargel | Fahrrad | asparagus | bicycle |
| Surfbrett | Kirsche | surfboard | cherry |
| Gameboy | Paprika | gameboy | pepper |
| Pfad | Zitrone | path | lemon |
| Pferd | Ananas | horse | pineapple |
| Spaten | Banane | spade | banana |
| Setzling | Löffel | seedling | spoon |
| Aprikose | Pinsel | apricot | brush |
| Peperoni | Tandem | chillies | tandem |
| Liane | Birne | liana | pear |
| Zwiebel | Handy | onion | cellphone |
| Kajak | Lauch | kayak | leek |
| Lupe | Mango | magnifier | mango |
| Axt | Olive | axe | olive |
| Tomate | Zange | tomato | pliers |
| Kiwi | Beil | kiwi | hatchet |
| Melone | Herd | melon | cooker |
| Apfel | Kamm | apple | comb |
| Hobel | Reis | plane | rice |
| Gurke | Bob | cucumber | bobsled |

**List 2 of the first action non-priming condition (cf. AC_1_ → A_2_, Fig. 1)**

| **German stimulus list** | | **English translation** | |
| --- | --- | --- | --- |
| **prime** | **target** | **prime** | **target** |
| Kronleuchter | Johannisbeere | chandelier | currant |
| Schmetterling | Inline-Skater | butterfly | inline skater |
| Fledermaus | Schreibfeder | bat | quill |
| Nummernschild | Korkenzieher | license plate | corkscrew |
| Gartenzwerg | Essstäbchen | garden gnome | chopstick |
| Kanaldeckel | Clementine | manhole cover | clementine |
| Litfaßsäule | Küchenwaage | advertising pillar | kitchen scales |
| Dachrinne | Haselnuss | gutter | hazelnut |
| Boden | Hammer | floor | hammer |
| Abzeichen | Regenschirm | badge | umbrella |
| Portrait | Tastatur | portrait | keyboard |
| Schildkröte | Heckenschere | turtle | hedge clippers |
| Pfosten | Frisbee | post | frisbee |
| Backstein | Gießkanne | brick | watering can |
| Türrahmen | Zwetschge | door frame | plum |
| Goldfisch | Rollschuhe | goldfish | rollerblades |
| Spatz | Messer | sparrow | knife |
| Torso | Garten | torso | garden |
| Efeu | Zither | ivy | zither |
| Strommast | Feldsalat | pylon | lamb's lettuce |
| Seehund | Spargel | seal | asparagus |
| Weinkiste | Surfbrett | wine crate | surfboard |
| Ameise | Gameboy | ant | gameboy |
| Lerche | Pfad | lark | path |
| Aushang | Pferd | announcement | horse |
| Elster | Spaten | magpie | spade |
| Glasstein | Setzling | glass stone | seedling |
| Maulwurf | Aprikose | mole | apricot |
| Terrasse | Peperoni | terrace | chillies |
| Krokus | Liane | crocus | liana |
| Schwan | Zwiebel | swan | onion |
| Enzian | Kajak | gentian | kayak |
| Palme | Lupe | palm | magnifier |
| Fink | Axt | finch | axe |
| Balken | Tomate | joist | tomato |
| Dach | Kiwi | roof | kiwi |
| Podest | Melone | podium | melon |
| Möwe | Apfel | seagull | apple |
| Logo | Hobel | logo | plane |
| Biber | Gurke | beaver | cucumber |

**List 2 of the second action non-priming condition (cf. A_1_ → AC_1_, Fig. 1)**

| **German stimulus list** | | **English translation** | |
| --- | --- | --- | --- |
| **prime** | **target** | **prime** | **target** |
| Schraubenzieher | Schildkröte | screwdriver | turtle |
| Schreibmaschine | Kronleuchter | typewriter | chandelier |
| Fleischklopfer | Schmetterling | meat tenderizer | butterfly |
| Schnittlauch | Nummernschild | chives | license plate |
| Staubsauger | Litfaßsäule | vacuum cleaner | advertising pillar |
| Kochlöffel | Kanaldeckel | cooking spoon | manhole cover |
| Malerwalze | Abzeichen | paint roller | badge |
| Wagenheber | Gartenzwerg | jack | garden gnome |
| Aubergine | Backstein | aubergine | brick |
| Blaubeere | Goldfisch | blueberry | goldfish |
| Brombeere | Dachrinne | blackberry | gutter |
| Kokosnuss | Weinkiste | coconut | wine crate |
| Kopfsalat | Glasstein | lettuce | glass stone |
| Mikroskop | Fledermaus | microscope | bat |
| Bumerang | Portrait | boomerang | portrait |
| Getreide | Pfosten | cereal | post |
| Himbeere | Strommast | raspberry | pylon |
| Peitsche | Türrahmen | whip | door frame |
| Pfirsich | Terrasse | peach | terrace |
| Webstuhl | Maulwurf | loom | mole |
| Fahrrad | Schwan | bicycle | swan |
| Kirsche | Aushang | cherry | announcement |
| Paprika | Balken | pepper | joist |
| Zitrone | Seehund | lemon | seal |
| Ananas | Podest | pineapple | podium |
| Banane | Torso | banana | torso |
| Löffel | Ameise | spoon | ant |
| Pinsel | Lerche | brush | lark |
| Tandem | Krokus | tandem | crocus |
| Birne | Spatz | pear | sparrow |
| Handy | Boden | cellphone | floor |
| Lauch | Dach | leek | roof |
| Mango | Elster | mango | magpie |
| Olive | Fink | olive | finch |
| Zange | Palme | pliers | palm |
| Beil | Enzian | hatchet | gentian |
| Herd | Möwe | cooker | seagull |
| Kamm | Efeu | comb | ivy |
| Reis | Biber | rice | beaver |
| Bob | Logo | bobsled | logo |

**List 2 of the sound priming condition (cf. S_2_ → S_1_, Fig. 1)**

| **German stimulus list** | | **English translation** | |
| --- | --- | --- | --- |
| **prime** | **target** | **prime** | **target** |
| Lautsprecher | Klapperschlange | loudspeaker | rattlesnake |
| Alarmanlage | Bohnermaschine | alarm system | floor polisher |
| Mundharmonika | Küchenmaschine | harmonika | kitchen machine |
| Trillerpfeife | Akkuschrauber | whistle | cordless screwdriver |
| Synthesizer | Kanarienvogel | synthesizer | canary |
| Dudelsack | Bohrmaschine | bagpipes | drill |
| Hubschrauber | Kuckucksuhr | helicopter | cuckoo clock |
| Wasserfall | Martinshorn | waterfall | siren (emergency vehicle) |
| Wellensittich | Kettensäge | budgerigar | chainsaw |
| Kontrabass | Klospülung | double bass | flush |
| Metronom | Nachtigall | metronome | nichtingale |
| Rotkehlchen | Eisenbahn | robin | railway |
| Schwein | Kopfhörer | pig | headphones |
| Ventilator | Mandoline | fan | mandolin |
| Akkordeon | Gewitter | accordion | thunderstorm |
| Piepser | Jagdhorn | bleeper | hunting horn |
| Elefant | Keyboard | elephant | keyboard (musical instrument) |
| Türklingel | Saxophon | doorbell | saxophone |
| Kuckuck | Triangel | cuckoo | triangle |
| Schnake | Klingel | crane fly | bell |
| Alphorn | Papagei | alphorn | parrot |
| Schaf | Violine | sheep | violin |
| Biene | Glocke | bee | bell |
| Posaune | Grille | trombone | cricket |
| Panflöte | Hummel | panpipe | bumblebee |
| Megaphon | Specht | megaphone | woodpecker |
| Frosch | Wecker | frog | alarm clock |
| Donner | Banjo | thunder | banjo |
| Mofa | Geige | moped | violin |
| Echo | Mücke | echo | mosquito |
| Hahn | Orgel | rooster | organ |
| Löwe | Radio | lion | radio |
| Cembalo | Ziege | cembalo | goat |
| Sirene | Eule | siren | owl |
| Pfeife | Huhn | pipe | hen |
| Oboe | Hund | oboe | dog |
| Cello | Hupe | cello | horn |
| Pauke | Rabe | kettledrum | raven |
| Krähe | Tuba | crow | tuba |
| Fön | Uhu | hairdryer | eagle owl |

**List 2 of the first sound non-priming condition (cf. SC_1_ → S_2_, Fig. 1)**

| **German stimulus list** | | **English translation** | |
| --- | --- | --- | --- |
| **prime** | **target** | **prime** | **target** |
| Schirmständer | Lautsprecher | umbrella stand | loudspeaker |
| Muskatnuss | Alarmanlage | nutmeg | alarm system |
| Destilliergerät | Mundharmonika | destillator | harmonika |
| Servierwagen | Trillerpfeife | trolley | whistle |
| Blumenbeet | Synthesizer | flowerbed | synthesizer |
| Basilikum | Dudelsack | basil | bagpipes |
| Bilderrahmen | Hubschrauber | picture frame | helicopter |
| Kuchenform | Wasserfall | cake pan | waterfall |
| Tollkirsche | Wellensittich | belladonna | budgerigar |
| Angelhaken | Kontrabass | fish hook | double bass |
| Isomatte | Metronom | sleeping pad | metronome |
| Artischocke | Rotkehlchen | artichoke | robin |
| Brille | Schwein | glasses | pig |
| Petersilie | Ventilator | parsley | fan |
| Knoblauch | Akkordeon | garlic | accordion |
| Spinat | Piepser | spinach | bleeper |
| Laterne | Elefant | lantern | elephant |
| Champignon | Türklingel | mushroom | doorbell |
| Limone | Kuckuck | lime | cuckoo |
| Rikscha | Schnake | rickshaw | crane fly |
| Magnet | Alphorn | magnet | alphorn |
| Kerze | Schaf | candle | sheep |
| Trage | Biene | stretcher | bee |
| Kissen | Posaune | pillow | trombone |
| Teleskop | Panflöte | telescope | panpipe |
| Quittung | Megaphon | receipt | megaphone |
| Dattel | Frosch | date | frog |
| Ingwer | Donner | ginger | thunder |
| Album | Mofa | album | moped |
| Lampe | Echo | lamp | echo |
| Zelt | Hahn | tent | rooster |
| Feld | Löwe | field | lion |
| Pfeiler | Cembalo | pillar | cembalo |
| Statue | Sirene | statue | siren |
| Lifter | Pfeife | lifter | pipe |
| Zimt | Oboe | cinnamon | oboe |
| Tuch | Cello | cloth | cello |
| Helm | Pauke | helmet | kettledrum |
| Couch | Krähe | couch | crow |
| Dill | Fön | dill | hairdryer |

**List 2 of the second sound non-priming condition (cf. S_1_ → SC_1_, Fig. 1)**

| **German stimulus list** | | **English translation** | |
| --- | --- | --- | --- |
| **prime** | **target** | **prime** | **target** |
| Klapperschlange | Destilliergerät | rattlesnake | destillator |
| Bohnermaschine | Tollkirsche | floor polisher | belladonna |
| Küchenmaschine | Bilderrahmen | kitchen machine | picture frame |
| Akkuschrauber | Schirmständer | cordless screwdriver | umbrella stand |
| Kanarienvogel | Servierwagen | canary | trolley |
| Bohrmaschine | Champignon | drill | mushroom |
| Kuckucksuhr | Blumenbeet | cuckoo clock | flowerbed |
| Martinshorn | Artischocke | siren (emergency vehicle) | artichoke |
| Kettensäge | Angelhaken | chainsaw | fish hook |
| Klospülung | Muskatnuss | flush | nutmeg |
| Nachtigall | Kuchenform | nichtingale | cake pan |
| Eisenbahn | Laterne | railway | lantern |
| Kopfhörer | Petersilie | headphones | parsley |
| Mandoline | Basilikum | mandolin | basil |
| Gewitter | Kissen | thunderstorm | pillow |
| Jagdhorn | Isomatte | hunting horn | sleeping pad |
| Keyboard | Limone | keyboard (musical instrument) | lime |
| Saxophon | Knoblauch | saxophone | garlic |
| Triangel | Dattel | triangle | date |
| Klingel | Pfeiler | bell | pillar |
| Papagei | Quittung | parrot | receipt |
| Violine | Teleskop | violin | telescope |
| Glocke | Album | bell | album |
| Grille | Ingwer | cricket | ginger |
| Hummel | Lifter | bumblebee | lifter |
| Specht | Rikscha | woodpecker | rickshaw |
| Wecker | Spinat | alarm clock | spinach |
| Banjo | Tuch | banjo | cloth |
| Geige | Lampe | violin | lamp |
| Mücke | Dill | mosquito | dill |
| Orgel | Zelt | organ | tent |
| Radio | Feld | radio | field |
| Ziege | Kerze | goat | candle |
| Eule | Helm | owl | helmet |
| Huhn | Trage | hen | stretcher |
| Hund | Brille | dog | glasses |
| Hupe | Statue | horn | statue |
| Rabe | Magnet | raven | magnet |
| Tuba | Couch | tuba | couch |
| Uhu | Zimt | eagle owl | cinnamon |
